# *In Silico* Discovery of Potential VEGFR-2 Inhibitors from Natural Derivatives for Anti-Angiogenesis Therapy

**DOI:** 10.3390/ijms150915994

**Published:** 2014-09-11

**Authors:** Jing Li, Nan Zhou, Kun Luo, Wei Zhang, Xinru Li, Chuanfang Wu, Jinku Bao

**Affiliations:** 1 College of Life Sciences and Key Laboratory for Bio-Resources of Ministry of Education, Sichuan University, Chengdu 610064, China; E-Mails: lijing_22@aliyun.com (J.L.); zhnanx@live.com (N.Z.); luokun9527@gmail.com (K.L.); zhgwe2010@163.com (W.Z.); lixinruswxxx@163.com (X.L.); wuchuanfang@gmail.com (C.W.); 2 State Key Laboratory of Biotherapy, West China Hospital, Sichuan University, Chengdu 610041, China

**Keywords:** anti-angiogenesis, drug, virtual screening, simulation, VEGFR-2

## Abstract

Angiogenesis is the growth of new capillaries from existing blood vessels that supply oxygen and nutrients and provide gateways for immune surveillance. Abnormal vessel growth in term of excessive angiogenesis is a hallmark of cancer, inflammatory and eye diseases. VEGFR-2 (vascular endothelial growth factor receptor 2) dominating the process of angiogenesis has led to approval of therapeutic inhibitors and is becoming a promising target for anti-angiogenic drugs. Notwithstanding these successes, the clinical use of current VEGFR-2 blockers is more challenging than anticipated. Taking axitinib as a reference drug, in our study we found three potent VEGFR-2 inhibitors (ZINC08254217, ZINC08254138, and ZINC03838680) from natural derivatives. Each of the three inhibitors acquired a better grid score than axitinib (−62.11) when docked to VEGFR-2. Molecular dynamics simulations demonstrated that ZINC08254217– and ZINC08254138–VEGFR-2 complexes were more stable than axitinib. Similar to bind free energy for axitinib (−54.68 kcal/mol), such for ZINC03838680, ZINC08254217, and ZINC08254138 was −49.37, −43.32, and −32.73 kcal/mol respectively. These results suggested these three compounds could be candidate drugs against angiogenesis, with comparable VEGFR-2 binding affinity of axitinib. Hence findings in our study are able to provide valuable information on discovery of effective anti-angiogenesis therapy.

## 1. Introduction

Angiogenesis is the growth of new capillaries from existing blood vessels, which plays a central role during ontogenetic development and is essential in various physiological processes within human body like tissue growth and repair [1]. On the other side, uncontrolled vessel growth contributes to diseases such as tumor growth and metastasis, inflammatory disorder, and eye disease [2]. Given the complicated process of angiogenesis which is mediated by a large number of molecules, it is noticeable that a single growth factor, vascular endothelial growth factor (VEGF), regulates this process so predominantly [3]. VEGF stimulates angiogenesis by signaling through VEGF receptors (VEGFRs) which are high-affinity receptors for VEGF and are three receptor tyrosine kinases (VEGFR-1, VEGFR-2, and VEGFR-3) [4]. VEGFR-2 is stronger than VEGFR-1 whose kinase activity is 10-fold weaker than VEGFR-2 [5,6]. VEGFR-3 regulates embryonic angiogenesis and lymphangiogenesis [7]. Consequently, VEGFR-2 is the prime receptor transmitting angiogenic signals and VEGFR-2 inhibitors can decrease angiogenesis by blocking the VEGFR-2 mediated signaling pathway. Thus, the blockade of VEFGR-2 signaling cascades is able to provide a promising approach leading to develop anti-angiogenesis therapy.

Natural products have been the single most productive source of leads for the development of drugs [8,9]. For thousands of years the use of traditional medicines has closely linked medicinal and natural products [10]. Natural products have produced a profound impact on drug development as a result of their diversity, target affinity, and specificity [11,12,13]. In fact, as the richest source of novel compound classes for biological researches, natural products have enormous potential in drug development and it is no doubt that they will continue to play significant roles in new drug discovery.

Based on recent advances in drug discovery, there has been an increase in the number of synthetic and natural compounds that are available for testing using biochemical and cellular assays. However, empirical screening for lead compounds as further therapeutics is always constrained by compounds that exist physically in the experiment [14]. As computational method has been increasingly important in modern drug discovery, indicating it is worth of application. Virtual screening extends the possibilities to seek hits from currently unavailable chemical collections that are obtained through purchase for biological activity test. Molecular docking-based virtual screening, using a target which is a protein with experimentally determined structure, has become an established method for lead discovery in various drug development projects by filtering large virtual libraries [15,16]. Molecular dynamics simulation is a computer simulation that gives an exploration of physical movements of atoms and molecules within a period of time. Complementary to virtual screening and molecular docking, molecular dynamics simulation has become a standard tool for understanding the physical basis of interactions between macromolecular (like protein) receptors and their small-molecule ligands in drug discovery [17]. Together with binding free energy calculation and binding free energy decomposition, molecular dynamics simulation enables one to evaluate the stability of receptor–ligand complexes. In addition, this simulation takes into account contributions of different amino acid residues.

According to advances in understanding of molecular mechanisms of angiogenesis, VEGF (receptor) has become the prime anti-angiogenesis drug target [18]. For example, approved drugs for cancer and eye diseases comprise anti-VEGF antibody (bevacizumab, ranibizumab, pegaptanib, * etc.*) and VEGFR inhibitors (sunitinib, sorafenib, pazopanib, * etc.*) [1,3]. However, limited efficacy and drug resistance of these angiogenesis blockers remain outstanding problems. For instance, bevacizumab is likely to induce severe intraocular inflammation [19]; in the treatment of metastatic renal cell carcinoma, sunitinib can cause many adverse events, including thrombopenia and hypertension [20].

Considering currently available medicines’ limitations, the development of small-molecule inhibitors that prevent the VEGFRs signaling is an attractive anti-angiogenic strategy, since the resulting inhibition is likely less toxic for long-term use than antibody therapeutics [21]. Therefore, for the sake of potent anti-angiogenesis treatment, we aim at discovering potential VEGFR-2 inhibitors from natural products using computer-aided approaches. The process involves four major steps. We first downloaded structural information of natural derivatives from ZINC [22]; Second, we filtered these molecules via virtual screening; Third, we scored the screening results by molecular docking to find candidate drugs; Lastly, we assessed potential drugs through molecular dynamics simulations.

## 2. Results and Discussion

### 2.1. Docking Accuracy

Docking is the process of identifying the best binding conformation for a ligand, within the active site of a receptor whose structure is known. The most straightforward means to validate the accuracy of specified parameters for docking is to redock the co-crystallized ligand back into the binding pocket of the receptor [23,24]. RMSD, root mean square deviation, is considered as a necessary condition to monitor how much the structure has deviated from its initial geometry [25]. If the RMSD between its docking pose and crystallographic orientation is less than 2.0 Å, the docking protocol is correct and can be used to dock other ligands to the receptor protein [26].

**Figure 1:**
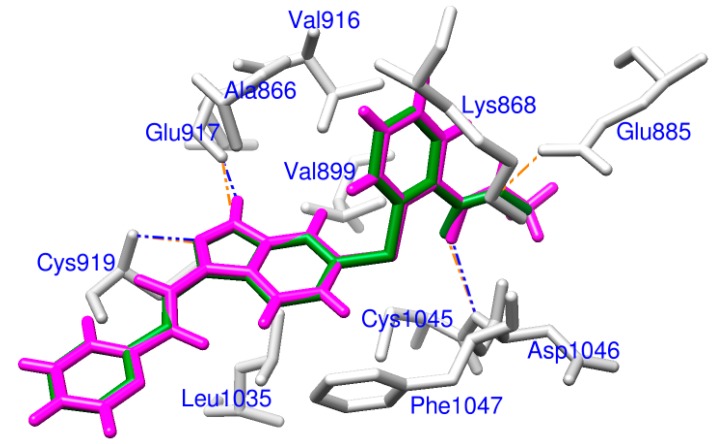
Docking validation, a comparison of the redocked binding mode (magenta) and the co-crystallized pose (forest green) of axitinib. Related residues of VEGFR-2 are labeled and shown as sticks in light gray. Hydrogen bonds are illustrated as dashed lines, blue for redocked mode and orange for co-crystallized pose.

Within the 4AG8 (*i.e.*, VEGFR-2) structure, a redocking manipulation was performed in our study to validate the docking methodology. We redocked the crystallized axitinib back to the binding site of VEGFR-2. For axitinib, the result revealed a grid score and heavy atom RMSD of −62.11 and 0.196 Å, respectively. It was confirmed that parameters for the DOCK program were rational for docking accuracy. As displayed in Figure 1, the redocked pose almost totally overlapped with experimental orientation. The subtle difference was that axitinib was redocked to VEGFR-2 forming three hydrogen bonds with Glu917, Cys919 and Asp1046, while one more hydrogen bond with Glu885 was observed in the 4AG8 structure. These results indicated DOCK could successfully redock the co-crystallized axitinib back into VEGFR-2 with high accuracy. So the docking protocol was credible for docking other natural derivatives to VEGFR-2.

### 2.2. Potential VEGFR-2 Inhibitors for Anti-Angiogenesis

After screening these 30,792 natural derivatives by evaluating score for each of them with vsLab, we sorted them in ascending sequence. The top ranked compound was the best and its grid score was −15.08. Derivatives with grid score(s) no more than −10 were considered as hits that could target VEGFR-2, which made up approximately 10% of 30,792 molecules including 3030 molecules in total. Then we docked these hits to the binding pocket of VEGFR-2 and compared them with the reference drug, axitinib. The docking results suggested that there were three potential drug-like derivatives (Table 1), ZINC08254217, ZINC08254138, and ZINC03838680, whose grid scores were −63.06, −62.59 and −62.34, respectively. Each of them got a grid score better than −62.11 of reference axitinib (a known VEGFR-2 inhibitor detailed in Section 3.1), according to which we selected them as candidate anti-angiogenic therapeutics.

**Figure 2:**
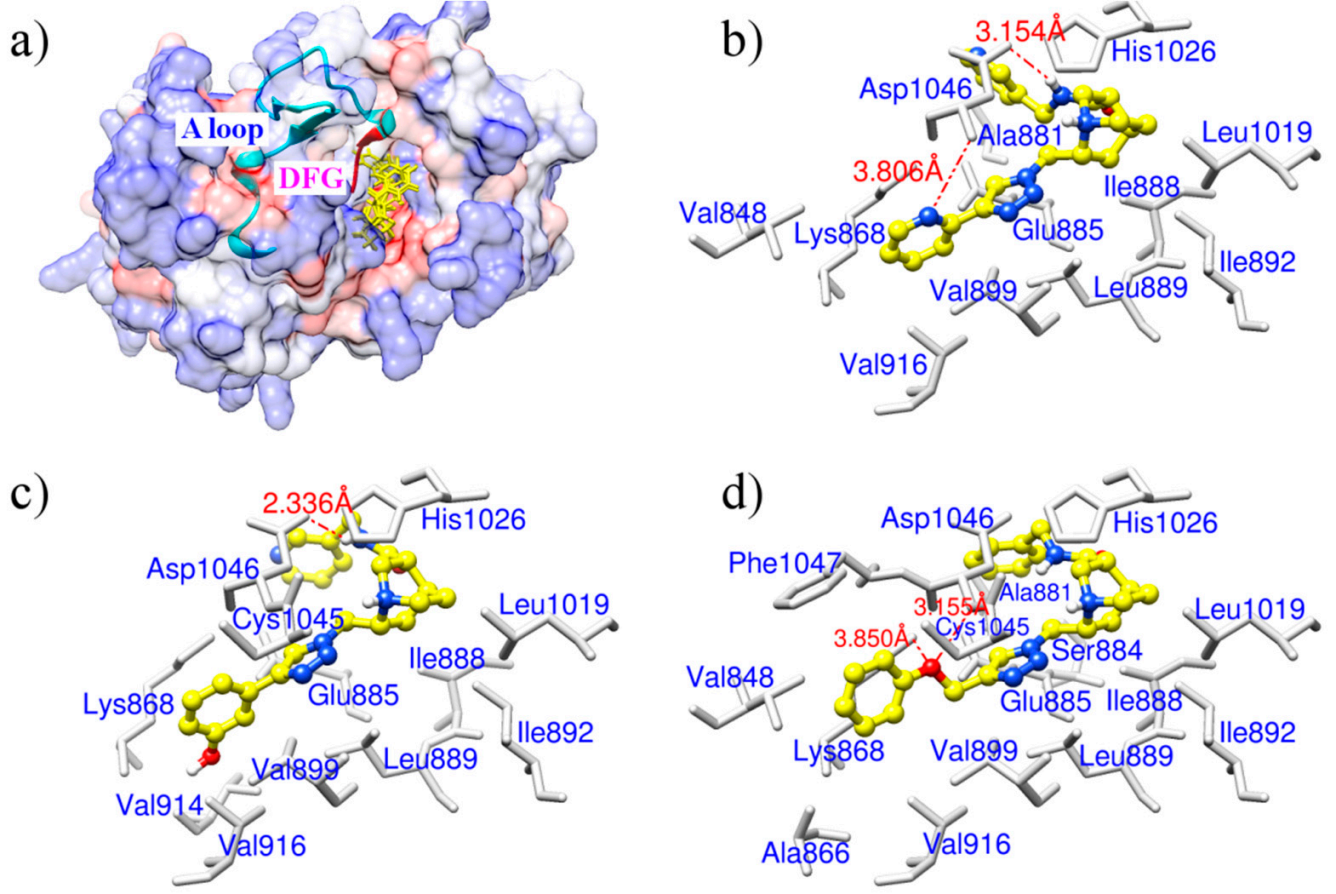
(**a**) An overview of binding modes for the three derivatives in the binding pocket of VEGFR-2. Derivatives are shown as yellow sticks. The surface of VEGFR-2 is colored to show hydrophobicity: from dodger blue for the most hydrophilic, to white, to orange red for the most hydrophobic. The activation loop (A loop) of VEGFR-2 is displayed as cartoon in cyan and the DFG motif of the activation loop is colored in red; (**b**) Binding mode for ZINC08254217; (**c**) Binding mode for ZINC08254138; (**d**) Binding mode for ZINC03838680. In (**b**–**d**), sticks in light gray are related residues of VEGFR-2, hydrogen bonds labeled with distance are shown as orange dashed lines, and derivatives are in shapes of ball and stick. Color codes for atoms of derivatives in (**b**–**d**): C is yellow, N is blue, and H is light gray.

These three derivatives were correctly docked into the binding pocket of VEGFR-2 as shown in Figure 2a. They shared similar docking modes with each other and were positioned in the hydrophobic pocket towards the DFG motif (Asp1046-Phe1047-Gly1048) of the activation loop, located between amino acid residues Asp1046 and Glu1075 [27]. We speculate this orientation may prevent the DFG motif from changing conformation, resulting in an inactive VEGFR-2 and subsequent reduced capacity to block angiogenesis. ZINC08254217 anchored VEGFR-2 forming two hydrogen bonds with the side chain of Asp1046 (Figure 2b). The two hydrogen bonds, with distance of 3.806 and 3.154 Å, stabilized the interaction between ZINC08254217 and VEGFR-2 in the activation loop. As shown in Figure 2c, the hydrogen bond with distance of 2.336 Å between ZINC08254138 and Asp1046 was observed in the interaction of ZINC08254138 and VEGFR-2. ZINC08254138 was sandwiched between Cys1045 and Glu885. This orientation made it steady in the protein–ligand interaction. The docking mode of ZINC03838680 was exhibited in Figure 2d. ZINC03838680 formed bidentate hydrogen bonds with side chain of Asp1046 and Lys868. The first aryl ring of ZINC03838680 stretched close to Ala881 and the second aryl ring oriented towards Lys868 with the help of the hydrogen bonds, enabling ZINC03838680 to contact more residues when interacted with VEGFR-2 at the binding site.

**Table 1:** Three candidate anti-angiogenic agents targeting VEGFR-2 result from virtual screening and molecular docking.

Compound	Structure	Grid Score
ZINC08254217	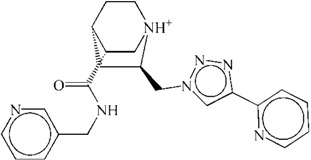	−63.06
ZINC08254138	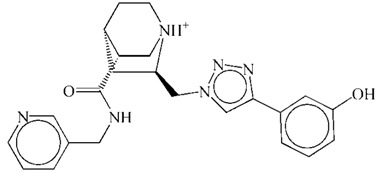	−62.59
ZINC03838680	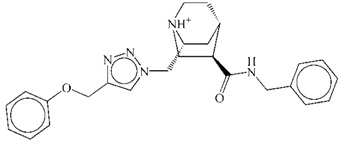	−62.34
Axitinib	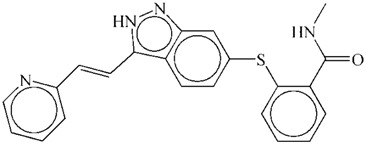	−62.11

In the 4AG8 structure, the reference drug (axitinib) bound to VEGFR-2 forming four hydrogen bonds as illustrated in Figure 1. Although the figure for formed hydrogen bonds of the three drug-like natural derivatives was two or three less than that of axitinib, they were comparable to axitinib in other respects. As shown in Figure 2, these three molecules were docked into the hydrophobic pocket formed by Val848, Val899, Val914, Val916, Leu889, Leu1019, Ile888, Ile892, Ala866, Ala881, Cys1045, and Phe1047. In addition to the strong hydrophobic interactions, the three derivatives were placed in the binding pocket of VEGFR-2 towards the DFG motif of the activation loop. It is known that the conformational change of the DFG motif improves the capacity of the protein tyrosine kinases to bind ATP and enables the phosphorylation of protein tyrosine kinases, resulting in the activation of kinases [28]. Thereby, VEGFR-2–ligand interaction would disrupt the allosteric change of this motif and consequently inhibit the kinase activity. Presumably, the lower, negative gird score and the appropriate binding modes of these derivatives indicated they could target VEGFR-2 with high affinity like axitinib. Therefore, these three derivatives could be used as potential VEGFR-2 inhibitors for anti-angiogenesis therapy.

### 2.3. Molecular Dynamics Simulation Analysis

Molecular dynamics simulation is an approach that could give a way to dynamically evaluate interactions between receptors and ligands within a specific time period. Therefore, we performed the 10 ns molecular dynamics simulations for VEGFR-2–inhibitor complexes to get in-depth understanding of these binding interactions.

**Figure 3:**
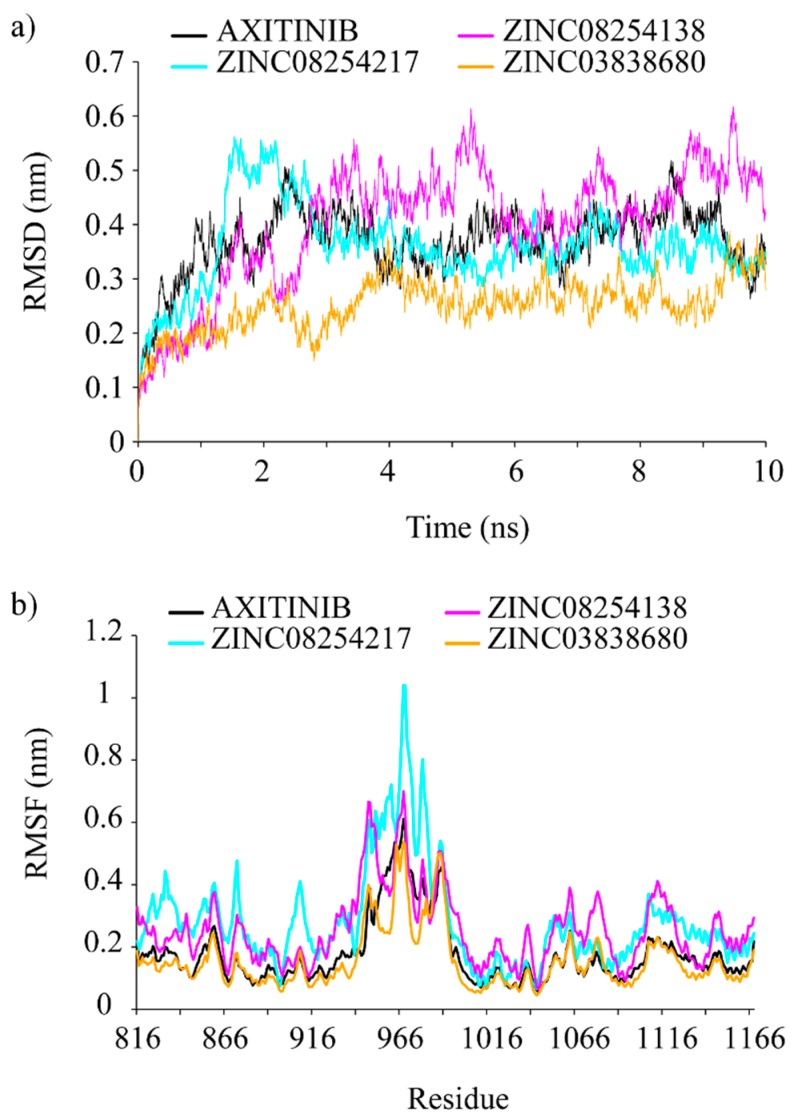
(**a**) Root mean square deviations (RMSDs) of backbone atoms of VEGFR-2. RMSD achieving a plateau after a short period of fluctuation indicates the receptor–ligand complex is stable. The lower the RMSD value, the more stable the system is; (**b**) RMSFs of backbone atoms of VEGFR-2. Flexible (high RMSF value) region contributes to structural changes while inflexible (low RMSF value) region stimulates ligand to bind the receptor. Both in (**a**) and (**b**), inhibitors within each VEGFR-2–inhibitor complex represented by a unique color: black for axitinib, cyan for ZINC08254217, magenta for ZINC08245138, and orange for ZINC03838680.

Putative VEGFR-2 inhibitors (ZINC08254217, ZINC08254138, and ZINC03838680) and the reference ligand (*i.e.*, axitinib) had backbone RMSD values ranging from 0.1 to 0.6 nm, as detailed in Figure 3a. It was obvious that all systems reached the platform at 4 ns except ZINC08254138 which did not reach equilibrium until 6 ns. Unlike other complexes waved again at 9 ns, the ZINC08254217 system kept stabilized till the end of the simulation. Similar to axitinib, the backbone RMSD of ZINC08254217–VEGFR-2 complex deviated by 0.4 nm when stabilized. After the equilibrium, the ZINC08254138 system fluctuated between 0.4 and 0.6 nm, and the ZINC03838680–VEGFR-2 complex fluctuated around 0.3 nm. Compared with the axitinib–VEGFR-2 system, the ZINC08254217– and ZINC03838680–VEGFR-2 complexes were more stable, which suggested that these two derivatives may have a stronger affinity towards VEGFR-2 when compared to axitinib. The ZINC08254138–VEGFR-2 complex equilibrated later and oscillated widely owing to weaker hydrogen-bonding interactions because there was only one hydrogen bond formed when bound to VEGFR-2 as shown in Figure 2c. Although the ZINC08254138 system cost longer to equilibrate and the RMSD values were greater than that of axitinib, the ZINC08254138–VEGFR-2 complex was still stable. Therefore, it was reasonable to believe that these three derivatives were better than or equal to axitinib.

RMSF denotes root mean square fluctuation, which is used as a means describing flexibility differences among protein residues in the molecular dynamics trajectory [29]. As illustrated in Figure 3b, the four systems shared similar backbone root mean square fluctuation (RMSF) profiles. Region Lys941–Met1016 was the most flexible, suggesting residues in it contributed less to the receptor–ligand interactions. This was consistent with the fact that residues of Thr940–Glu989 had been approved without noticeable impacts on VEGFR-2’s catalytic activity [27]. Region Ala866–Lys941 and region Met1016–Arg1066 were more stable than other regions, indicating residues in them were pivotal for the binding of the proposed inhibitors to VEGFR-2. The two zones were relatively conserved hydrogen interaction regions [30]. Important residues like Cys917, Cys919 and Asp1046 were in them. These residues could form hydrogen bonds with inhibitors and thus made these regions less flexible. The formation of hydrogen bonds between Asp1046 and the three natural derivatives were also observed in our study, contributing to show similar RMSF profiles. Compared with the axitinib–VEGFR-2 complex, the ZINC03838680 system was more stable but the ZINC08254217– and ZINC08254138–VEGFR-2 complexes were a little flexible. These results from molecular dynamics simulations demonstrated that regardless of insignificant differences between these inhibitors, all the three derivative molecules (ZINC08254217, ZINC08254138, and ZINC03838680) were comparable to axitinib and could target VEGFR-2 effectively against angiogenesis.

### 2.4. Binding Free Energy and Its Decomposition

With the objective of further exploring the interactions between VEGFR-2 and the four inhibitors, binding free energy calculations followed by binding free energy decomposition were performed using MM/GBSA method in Amber 9. Table 2 listed the binding free energy with every component for each VEGFR-2 inhibitor. For every protein–ligand interaction, the gas phase energy (∆*G*_gas_) contributed to the binding while the solvation free energy (∆*G*_solv_) opposed binding. Scrutinizing the gas phase terms, we found that the van der Waals interactions (∆*E*_vdw_) were more important than the electrostatic interactions (∆*E*_ele_), especially for associations between VEGFR-2 and three potential anti-angiogenic derivatives. While van der Waals contributions of three derivatives were as strong as that of axitinib, their electrostatic contributions were much weaker. Although solvation free energies were unfavorable for interactions, their nonpolar contributions (∆*E*_surf_) promoted ligand binding. Unfavorable solvation free energies mainly resulted from polar solvation free energies (∆*E*_GB_) that were positive unfavorable and much stronger than slightly favorable nonplar terms. With thorough comparison of binding free energies between these inhibitors, axitinib, the known VEGFR-2 inhibitor, had the lowest binding free energy (−54.68 kcal/mol). Similarly, ZINC03838680, ZINC08254217, and ZINC08254138 with binding free energies of −49.37, −43.32, and −32.73 kcal/mol hinted they could bind to VEGFR-2 like axitinib.

**Figure 4:**
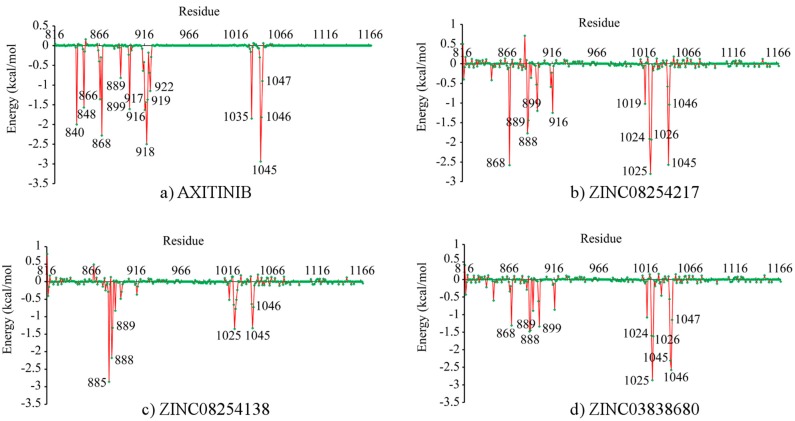
Binding free energy decomposition on a per-residue basis for each VEGFR-2–inhibitor complex. (**a**) Axitinib; (**b**) ZINC08254217; (**c**) ZINC08254138 and (**d**) ZINC03838680. For each residue, the lower the energy, the more important the residue in the receptor–ligand binding.

The binding free energy decomposition revealing contributions of each residue during interaction was illustrated in Figure 4. As demonstrated in Figure 4, four inhibitors had similar interaction patterns. Important residues were observed in two significantly different regions, which was consistent with findings from RMSF results in Figure 3b. This may result from different domains of the glycine-rich nucleotide binding loop for ATP binding (Gly841–Gly846) and the activation loop for phosphorylation (Asp1046–Glu1075) in VEGFR-2 [4,27]. For axitinib binding, key residues were Leu840, Val848, Ala866, Lys868, Leu889, Val899, Val916, Glu917, Phe918, Cys919, Gly922, Leu1035, Cys1045, Asp1046, and Phe1047. Instead of Leu1035, residues around Ile1025 were critical for derivatives binding but not axitinib. This was because the three derivatives were far from Leu1035 except that axitinib was steric accessible as displayed in Figure 1. Relative to axitinib, Cys1045 and Asp1046 were less essential to the ZINC08254138–VEGFR-2 complex and contributions of residues around Ile888 were less important for the ZINC03838680–VEGFR-2 complex. No matter how different in details, these three derivatives, especially ZINC08254217, had similar key residues when bound to VEGFR-2. These findings revealed that like axitinib, the three drug-like derivatives could also target VEFRR-2 with similar profiles, consequently terminating angiogenesis.

**Table 2:** The predicted binding free energy (kcal/mol) for each VEGFR-2 inhibitor via MM/GBSA method.

Terms ^a^	AXITINIB	ZINC08254217	ZINC08254138	ZINC03838680
∆*E*_ele_	−36.32 ± 3.02	−8.22 ± 13.26	−16.24 ± 23.84	−15 ± 10.28
∆*E*_vdw_	−56.19 ± 2.87	−54.91 ± 2.58	−41.37 ± 4.6	−59.54 ± 2.88
∆*G*_gas_	−92.51 ± 2.95	−63.14 ± 13.4	−57.61 ± 25.96	−74.54 ± 10.32
∆*E*_surf_	−6.28 ± 0.11	−6.95 ± 0.16	−6.04 ± 0.35	−7.5 ± 0.23
∆*E*_GB_	44.11 ± 1.79	26.76 ± 12.38	30.91 ± 22.35	32.67 ± 10.06
∆*G*_solv_	37.83 ± 1.8	19.81 ± 12.4	24.88 ± 22.18	25.17 ± 10.12
∆*G*_bind_	−54.68 ± 2.62	−43.32 ± 3.36	−32.73 ± 5.81	−49.37 ± 3.21

^a^ Terms: ∆*E*_ele_, electrostatic contribution; ∆*E*_vdw_, van der Waals contribution; ∆*E*_int_, internal contributions including bond, angle, and torsion terms, was not listed because its value was 0 for every inhibitor; ∆*G*_gas_, gas phase free energy; ∆*E*_surf_, nonpolar solvation contribution; ∆*E*_GB_, polar solvation contribution; ∆*G*_solv_, solvation free energy; ∆*G*_bind_, binding free energy. ∆*G*_bind_ = ∆*G*_gas_ + ∆*G*_solv_; ∆*G*_gas_ = ∆*E*_ele_ + ∆*E*_vdw_; ∆*G*_solv_ = ∆*E*_surf_ + ∆*E*_GB_.

### 2.5. Anti-Angiogenic Use of these VEGFR-2 Inhibitors

Chronic skin psoriasis is a kind of inflammatory disease that affects approximately 3% of the world population [31]. Currently available treatments for psoriasis are topical therapy, phototherapy, systematic therapy, and biologic therapy [32]. They are not effective for everyone and may cause serious side effects. For instance, long-term use of corticosteroids (topical therapy) can cause skin to become thinner; phototherapy utilizing ultraviolet may lead to skin cancer if use too much; male patients taking methotrexate (systematic therapy) should not try for babies because it can damage the developing baby; some biologic drugs (infliximab, etanercept, adalimumab) are associated with increased incidence of lymphoma [33,34]. Angiogenesis is increasingly found to contribute to psoriasis development but has not received enough attention [35]. There still have no VEGFR inhibitors licensed for psoriasis therapy. Remarkably, VEGFR inhibitors like sunitinib and sorafenib were found to be effective against psoriasis [36,37]. Therefore, these potential VEGFR-2 inhibitors may be able to provide a promising approach for future psoriasis treatment, alleviating patients’ suffering from psoriasis and improving their quality of life.

It is also thought that angiogenesis is necessary for tumor growth and metastasis. In fact, tumor vessels are dysfunctional and every layer of the tumor vessel wall is abnormal [38]. Angiogenesis contributes to cancer development and angiogenic feedback from tumors then render nearby normal cells excessive angiogenesis. The reference drug axitinib has been approved for various malignancies, including renal cell carcinoma, thyroid cancer, non-small cell lung cancer, and melanoma [39]. Hence the three comparable VEGFR-2 inhibitors discovered in our study are speculated to prevent or slow the growth of such malignancies. Apart from cancer and inflammatory diseases, angiogenesis is also a pathogenic factor for pulmonary hypertension and eye diseases [40]. That is to say these VEGFR-2 inhibitors may function as drugs for those disorders as well.

## 3. Experimental Section

### 3.1. Data Collection and Preparation

Natural derivatives used as ligands for VEGFR-2 were downloaded from ZINC database [22]. ZINC, a free database, contains more than 35 million purchasable compounds in ready-to-dock, 3D (three dimensional) formats for virtual screening. Znd (ZINC natural derivatives) is a special subset consisting of chemically modified natural products last updated on 21 February 2013, with 30,792 items in total. On 8 February 2014, we downloaded all compounds contained in Znd as ligands for the discovery of potential anti-angiogenic drugs targeting VEGFR-2.

The RCSB protein data bank (PDB) is an archive containing information about experimentally determined structures of proteins [41]. We downloaded the structure of receptor tyrosine kinase VEGFR-2 with id 4AG8 (PDB: 4AG8) [42]. VEGFR-2 in the crystal structure is in complex with axitinib, an angiogenic inhibitor approved by FDA (U.S. Food and Drug Administration) on 27 January 2012 [43]. Axitinib is a VEGFRs-specific inhibitor with minimal adverse reactions and enhanced safety [39]. Thus, axitinib is also used as a reference drug for the discovery of potential VEGFR-2 inhibitors in our study.

Chimera was used to extract the receptor VEGFR-2 and the ligand axitinib from the 4AG8 structure [44]. Missing residues and atoms of VEGFR-2 were rebuilt with the SWISS-MODEL software [45]. The preparation of protein was completed by dock prep tool from Chimera: extraneous atoms were stripped, the method of also considering H-bonds was used to add hydrogen atoms, protonation states for histidine were detected automatically, amber ff99sb force field was used to assign charges for standard residues [46], and other residues were handled with AM1-BCC methods [47]. The co-crystallized axitinib was modified by adding charges using add charge tool from Chimera. No further modifications were needed for derivatives because compounds from ZINC were already in the correct format and could be used in docking studies directly [22].

### 3.2. Virtual Screening

The virtual screening approach in drug discovery has been used to seek many hits for a receptor protein by reducing the massive chemical library of structures in the direction of the target [48]. We first downloaded structural information of natural derivatives from ZINC and then we filtered them via virtual screening. We used vsLab to simulate the virtual screening campaign searching for lead compounds from these downloaded natural derivatives [49]. As an extension of VMD program [50], vsLab utility is an easy-to-use graphical interface which invokes AutoDock to ensure the reliability and accuracy of screening results [51]. VEGFR-2 for the virtual screening process was optimized by AutoDockTools [51], water was removed and hydrogen was added. The grid box was located at the binding pocket with size 81 Å × 61 Å × 64 Å for VEGFR-2. Other parameters in vsLab were used as default. VsLab addressed the screening process by calculating score for each derivative. When completed, scored molecules were sorted in ascend order for the selection of lead derivatives.

### 3.3. Molecular Docking

Normally, docking is the identification of the best binding modes of a small molecule, or ligand, within the active site of a macromolecule, or receptor, whose structure is known. We used the UCSF DOCK suite (version 6.5) to perform the protein–ligand docking [52]. The DOCK suite of programs is designed to find favorable orientations of a ligand in a receptor. The molecular surface of the receptor was computed with a probe radius of 1.4 Å. Spheres were generated from the molecular surface with a maximum and minimum radius of 4.0 and 1.4 Å, respectively. The subset of spheres representing the binding site for VEGFR-2 was identified automatically as the location of the co-crystallized axitinib within 5.0 Å RMSD from every atom of the crystal structure of the ligand. The location and size of the grid box was defined enclosing 5 Å extra margins added in all six directions. The grid-based energy scoring function was used to calculate molecular mechanics interaction energies, consisting of van der Waals and electrostatic components, to generate necessary grid files for rapid score evaluation. The flexible ligand docking in which ligands were allowed to be flexible was implemented at last to evaluate docked derivatives. The grid score, * i.e.*, binding probability for each natural derivative was calculated by structurally rearranging them in response to VEGFR-2.

### 3.4. Molecular Dynamics Simulation

Molecular dynamics simulation has been widely used to study the formations, structures and interactions between molecules in a system [53]. The main objective in conducting molecular dynamics simulations is to produce a trajectory of molecules within a given time period. All molecular dynamics simulations were carried out by GROMACS package (version 4.5) [54]. We first processed the protein structure with tip3p water mode and AMBER99SB force field generating coordinates and topology files [55,56]. Then we dealt with the ligand and acpype was used to create ligand’s topology [57]. Following the completion of the preparation of the protein and ligand, we built the complex and topology which were in agreement with respect to the content of the system. Then we defined the unit cell in shape of dodecahedron and filled it with simple point charge (SPC) water molecules [58]. The distance between the solute and the box was set to 1.0. To neutralize the solvated system, we added ions (Na^+^ and Cl^−^) with salt concentration of 0.15 mol/L. After that, we relaxed the assembled system via energy minimization using steepest descent minimization algorithm until the maximum force under 1000 kcal/mol/nm. In addition, then we equilibrated the protein–ligand complex with two steps. The first step was the 100 ps (picosecond, or 10^−12^ of a second) NVT ensemble (constant Number of particles, Volume, and Temperature) to stabilize the system at 300 K and the second step, the NPT (constant Number of particles, Pressure, and Temperature) equilibration within 100 ps, stabilized the system’s pressure using coupling reference pressure of 1 bar. After the two equilibration steps, the system was well optimized and at last, we ran a 10 ns molecular dynamics simulation with a time step of 2 fs (femtosecond, or 10^−15^ of a second). Throughout the simulation process, energy and trajectory information were collected every 2 ps, long-range electrostatics was investigated by particle mesh Ewald (PME) method [59], and all bond lengths were limited by LINCS algorithm [60].

### 3.5. Energy Analysis via MM/GBSA Approach

Free energy calculation is a detailed investigation of energetic factors that are responsible for molecular stability or binding affinity [61]. It has become a powerful tool in providing quantitative measurement of protein-ligand interactions [62]. The Molecular Mechanics/Generalized Born Surface Area (MM/GBSA) is a widely exploited method which applies no empirical parameters in its free energy calculation [63]. In MM/GBSA, the binding free energy (∆*G*_bind_) of receptor (R) + ligand (L) → complex (RL) is calculated as [64],

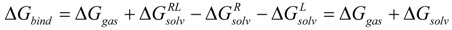
(1)


∆*G*_gas_ is the interaction energy between R and L in the gas phase and ∆*G*_solv_ includes solvation free energies of R, L and RL. We performed the MM/GBSA calculation by mm_pbsa.pl of AMBER 9 [65]. The binding free energy was decomposed into three terms, including a gas phase energy, a solvation free energy and an entropy term, as shown below [66],

∆*G*_bind_ = ∆*H*_gas_ −*T*∆*S* + ∆*G*_solv_ ≈ ∆*E*_gas_ + ∆*G*_solv_ −*T*∆*S* (2)

If ligands have similar structures and binding modes, it is acceptable to exclude the entropy contribution (−*T*∆*S*) in practice [64,67]. Then the binding free energy is evaluated by [61]:

∆*G*_bind_ = ∆*E*_gas_ + ∆*G*_solv_ = ∆*E*_MM_ + ∆*G*_GBSA_ (3)

∆*E*_gas_ = ∆*E*_MM_ = ∆*E*_int_ + ∆*E*_ele_ + ∆*E*_vdw_ (4)

∆*G*_solv_ = ∆*G*_GBSA_ = ∆G_GB_ + ∆*G*_SA_ (5)

∆*E*_gas_, the complete gas phase force field energy, is the molecular mechanics (MM) part, including internal energies (∆*E*_int_ comprising bond, angle, and torsion terms), electrostatic (∆*E*_ele_), and van der Waals (∆*E*_vdw_) energies. ∆G_GB_ is the polar solvation free energy evaluated via implicit solvation model Generalized Born (GB). The nonpolar solvation free energy (∆*G*_SA_) is estimated by solvent accessible surface area (SASA).

We first extracted the last 5 ns trajectory from the 10 ns simulation result and converted it to the crd format of AMBER 9 through VMD. Then a total of 100 snapshots were evenly created from the trajectory and finally, the single trajectory protocol was applied to calculate the MM/GBSA binding free energy. During the process, the exterior dielectric constant was set to 80, and the solute dielectric constant was set to 1.

Additionally, we took further advantage of MM/GBSA handling free energy decomposition. At the atomic level, it was made to analyze individual energy contributions of each residue to the total binding free energy and contributions of side chain and backbone as well [68]. The residue–ligand interaction consists of the gas phase energy (∆*G*_gas_) and solvation free energy (∆*G*_solv_), giving an insightful understanding of the investigated complex [69,70].

## 4. Conclusions

In this study, using axitinib as a reference drug, we found three potential VEGFR-2 inhibitors from natural derivatives through virtual screening and molecular docking analyses. The RMSD analysis for each VEGFR-2–inhibitor complex suggested these potential drugs could target VEGFR-2. Low binding free energy for each VEGFR-2–inhibitor interaction indicated the three derivatives had a strong affinity towards VEGFR-2. RMSF and binding free energy decomposition analyses illustrated similar contributing profiles of residues for interactions between VEGFR-2 and inhibitors. Results of molecular dynamics simulations demonstrated that similar to axitinib, these candidate drugs from natural derivatives could target VEGFR-2 effectively. Thus, we propose three inhibitors, listed in Table 1, for anti-angiogenesis therapy, based on their putative capacity to inactivate VEGFR-2 and subsequently reduce angiogenesis. To be honest, to what extent they would be approved for clinical use and to what extent they are safe still need further investigation as a lack of * in vitro* and *in vivo* experiments in our *in silico* study.
